# Potentiometric Determination of Chlorate Impurities in Hypochlorite Solutions

**DOI:** 10.1155/2019/2360420

**Published:** 2019-05-02

**Authors:** Dmitry V. Girenko, Al'ona A. Gyrenko, Nikolai V. Nikolenko

**Affiliations:** Ukrainian State University of Chemical Technology, Gagarin ave. 8, 49005 Dnepropetrovsk, Ukraine

## Abstract

The method of iodometric determination of chlorates impurities in sodium hypochlorite solutions for medical and veterinary purposes was developed. This method does not require sophisticated equipment and can be implemented directly where the solutions are used. The method is based on the different rates of interaction of ClO^−^ and ClO_3_^−^ with iodide ions depending on the acidity of the medium. We have shown that blank titration is advisable to improve the accuracy of the determination of low concentrations of chlorates in the matrix of hypochlorite which is present in excess since in this case possible systematic errors due to the presence of oxidizing impurities in the reagents are prevented. To quantify the low concentrations of chlorates, we proposed to remove 85-95% of hypochlorite ions by means of reducing their excess with sodium sulfite at pH 10.5. The solution of sodium sulfite does not require standardization before each analysis in the proposed procedure. The possibility of quantitative determination of chlorate impurities in the range of 2-50 mg/L in the presence of 50-500–fold excess of sodium hypochlorite with an error of 5% has been proved. The expanded uncertainty of chlorate determination did not exceed 0.6 mg/L.

## 1. Introduction

In modern medicine the solutions of sodium hypochlorite are widely used as antiseptic agents for external application, as well as for direct detoxication of the body when intravenously introduced. Sodium hypochlorite solutions display high biological activity against many Gram-positive and Gram-negative bacteria, most pathogenic fungi and viruses. The solutions are not toxic, do not cause allergic reactions, and the products of their transformations do not accumulate in the body of humans and animals [1–3]. Synthesis of hypochlorite solutions is characterized by low cost and can be conducted in the required amount directly at the place of their consumption, for example, in the zone of natural disasters or military conflicts.

Chemically pure solutions of sodium hypochlorite with pH of 7.5-8.5 are conveniently obtained by electrolysis of an isotonic NaCl solution. However, in the process of synthesis, as well as during long-term storage, chlorites and chlorates accumulate in solutions. They form due to disproportionation and interaction with impurities in the solution, especially with iron-group metal ions which act as catalysts for the decomposition of hypochlorite [4, 5]. The rate of accumulation of chlorites and chlorates during storage depends on many factors: a solution composition, pH, temperature, the action of light, and a container material. The most undesirable impurity in sodium hypochlorite solutions for medical purposes is chlorate ions [6]. Acting as blood poison, chlorates transfer hemoglobin to methemoglobin and cause the disintegration of red blood cells [7, 8].

Obviously, for the safe use of hypochlorite solutions in medicine and veterinary medicine, it is necessary to monitor their quality both at the synthesis stage and during long-term storage. As it is known [9], the analysis of such mixtures is among the most difficult analytical problems. In the solutions of sodium hypochlorite obtained electrolytically, the chlorate ions content is at least one or two orders of magnitude less than the hypochlorite ions content. For example, in the typical solutions for medicine, the NaClO content is 400-1500 mg/L (5·10^−3^–2·10^-2 ^mol/L), and NaClO_3_ - is not more than 40 mg/L (4·10^-4 ^mol/L).

Most of the existing methods for determining low levels of chlorate in sodium hypochlorite solutions are based on the use of liquid chromatography [10], spectrophotometry [11], and amperometry [12]. These methods require the availability of special equipment, which limits the possibility of their implementation in small production, pharmacies, and in the place where the end products are used.

More available are methods based on potentiometric titration. Among well-known methods is sequential potentiometric titration of hypochlorite, chlorite, and chlorate with a solution of arsenic (III) in the presence of an osmium tetroxide catalyst [13]. Such methods have a significant disadvantage, the toxicity of the titrant and the high cost of the catalyst. The alternative is potentiometric iodometric titration [9, 14]. The method is based on the reduction reactions of ClO^−^, ClO_2_^−^ and ClO_3_^−^ ions with iodide:

(1) ClO−+2I−+2H+→Cl−+I2+H2O,

(2) ClO2−+4I−+4H+→Cl−+2I2+2H2O,

(3) ClO3−+6I−+6H+→Cl−+3I2+3H2O.

The rates of these reactions depend, to a large degree, on the pH of the medium [9, 14]. In the medium of 0.5 M acetic acid the highest reduction rate is observed for hypochlorite ions. ClO_2_^−^ ions release iodine from iodide under these conditions extremely slowly, and ClO_3_^−^-ions do not react with iodide at all. In solutions of 1-4 M sulfuric acid, chlorate ions, in contrast to hypochlorite and chlorite ions, are reduced by iodide relatively slowly. Therefore, a titration under these conditions makes it possible to determine the total content of ClO^−^ and ClO_2_^−^-ions. In 6-8 M solutions of HCl all the reactions of ClO^−^, ClO_2_^−^ and ClO_3_^−^ ions with iodide ions proceed quantitatively and relatively quickly. Therefore, a titration in hydrochloric acid medium gives the total content of all studied oxycompounds of chlorine.

The method [15] enables to determine the content of sodium chlorate at the level of 1.0 mg/L with an error of 1.0% in drinking water in the absence or with a slight excess of active chlorine. The method [14] enables to determine sodium chlorate at the level of 50-150 mg/L in a matrix of 1-3 M sodium hypochlorite. It is based on a rather complex quantitative masking of hypochlorite in the medium of borate buffer at pH 10.5 by a potentiometric titration with sodium sulfite with further removal of its excess by means of triiodide I_3_^–^ and finally I_2_ formed is removed with thiosulfate. Then the sequential determination of ClO_2_^−^ and ClO_3_^−^ is carried out either by iodometric titration at the proper pH or by ion chromatography. The main disadvantage of the procedure is the use of sodium sulphite solution as a titrant, which decomposes with the rate of 0.1-0.15%/h due to interaction with oxygen.

Testing the iodometric methods showed that they do not assure the necessary accuracy of the analysis when determining low concentrations of chlorate (less than 40 mg/L) in a matrix of sodium hypochlorite excess (500-1500 mg/L). In this connection, the task was set to develop a relatively simple iodometric method for determining the impurities of chlorates in solutions of sodium hypochlorite for medical and veterinary purposes.

## 2. Materials and Methods

Initial solution of sodium hypochlorite (0.5 mol/L) was obtained by passing Cl_2_ through 2 M NaOH solution cooled to 0°C [16]. It was used as standard solution after determining sodium hypochlorite concentration according to the standard iodometric method [17]. A potentiometric titration was carried out using a microplatinum indicator electrode and a silver chloride reference electrode.

Solution of sodium thiosulfate was prepared from fixanal. For the preparation of standard solutions of NaClO_3_, Purissimum special reagent grade was used.

Evaluation of the instrumental uncertainty of the developed method for determination of sodium chlorate concentration was carried out according to [18].

## 3. Results and Discussion

### 3.1. Iodometric Determination of Chlorate Ions in Standard ClO^−^ Free Solutions

According to [14], the highest rate of reaction (3) occurs when iodide ions are in excess and acidity of the solutions is high. However, under such conditions the iodide ion is easily oxidized by air oxygen and, in the case of low chlorate concentrations, the reaction time can reach 20 minutes [13].

To assess the effect of dissolved oxygen on titration results, we studied the kinetics of this process in solutions free from ClO_3_^−^ (a similar volume of water or solution of 9 g/L NaCl was used instead of a sample of sodium chlorate solution). It was found that if the reaction mixture is in a closed flask with a volume of not more than 100 mL and without stirring, during the first 5 minutes the contribution of oxidation of iodide ions by air oxygen can be neglected. However, during this time the reaction does not complete which introduces an error in the quantitative determination.

The reaction of chlorate with iodide was carried out in the presence of bromide ions to increase its rate, as it was shown in [9]. The experiment on titration of sodium chlorate solutions with potassium bromide additives confirmed the possibility of the enhancement of the reaction (3) rate in the presence of excess bromide. At the same time, the results of the analysis are affected not only by the addition of KBr, but also by the sequence of mixing of the reagents. The least error in the determination of chlorates was obtained using the following procedure: 10.0 mL of concentrated hydrochloric acid (10-11 M) was added to a sample of 0.5 g of KBr, then 2.00 mL of the standard NaClO_3_ solution and after that in 1 min 2.5 mL of 10% KI was added. In 4 minutes amount of I_3_^–^ formed was determined by titration with 0.0025 N solution of sodium thiosulfate. An example of the obtained results is shown in Table 1. It is important to note that this method requires the aliquots of not more than 2,50 mL of chlorate containing sample.

In this sequence of analysis, first bromide ions react rapidly with chlorate ions in a strongly acid medium, and then the released bromine quantitatively oxidizes iodide ions to iodine:

(4) ClO3−+6Br−+6H+→3Br2+Cl−+3H2O,

(5) 3Br2+6I−→6Br−+3I2.

When adding bromide, there is no need to isolate the reaction mixture from air oxygen, for example, using hexane and nitrogen, as suggested in [10].

A blank titration showed that the accuracy of the method is affected by the purity of the reagents. In a number of cases, the use of reagents (KI, HCl, KBr) of p.a. from different manufacturers led to the overstated values of NaClO_3_ concentrations. Formation of additional amounts of iodine is explained by the presence of microimpurities of iodate and Fe(III) in the reagents. It is experimentally confirmed that the use of p.a. and puriss. reagents can lead to an error in the determination of chlorates from +2 to +5 mg/L.

The reagent blank correction should be obligatory part of microanalysis of ClO_3_^–^ impurities to compensate for sources of determinate error due to traces of oxidants and reducers in reagents and solvent. A blank titration enables to find the number of equivalents of oxidizing impurities *n*_*imp*_ = [*V*_*BC*_ · *C*(*Na*_2_*S*_2_*O*_3_)], where n_imp_ is the equivalent amount of impurities, which is determined by blank titration, mmol and V_BC_ is the equivalence point volume of sodium thiosulfate for the blank correction titration, mL.

An example of the results of titration of standard solutions with a reagent blank correction is given in Table 2. In the blank experiment, the test sample was replaced with double distilled water (2.00 mL). As follows from the comparison of the data, it is natural that the reagent bank correction increased the accuracy of the analysis.

**Figure 1:**
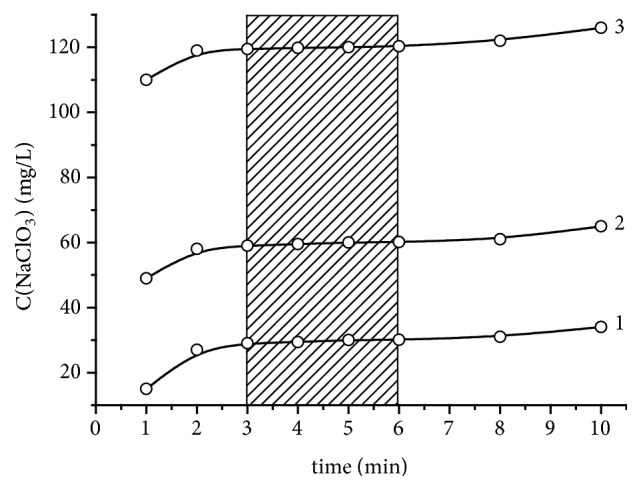
The dependence of the determined concentration of NaClO_3_ in the standard solution on the reaction time before titration with blank correction. The standard solution, mg/L: 30.0 (curve 1), 60.0 (curve 2), 120.0 (curve 3).

Figure 1 shows the results of the analysis of standard solutions of NaClO_3_ with the concentration of 30, 60, 120 mg/L in the presence of KBr depending on the time between the introduction of an aliquot of 10% KI into the reaction mixture and the titration. The figure shows that the optimal time is 3-6 minutes. Any increase in the time leads to a slight increase in the detected concentration which is associated with the additional oxidation of I^–^ by atmospheric oxygen.

**Figure 2:**
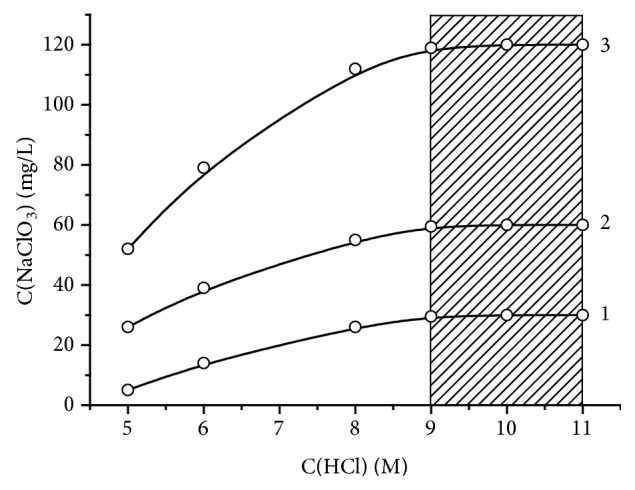
The dependence of the determined concentration of NaClO_3_ in a standard solution on the concentration of HCl with the reaction time before titration of 4 min. The standard solution, mg/L: 30.0 (curve 1), 60.0 (curve 2), 120.0 (curve 3).

It should be noted that the accuracy of the method for determining chlorate is significantly dependent on the concentration of hydrochloric acid required for the analysis. A preliminary experiment showed that the optimal concentration of hydrochloric acid for this analysis is 9.0-11.0 M (Figure 2). Thus, the final concentration of HCl in the reaction mixture after adding the sample and KI solution should be at least 5.5 M.

### 3.2. Iodometric Determination of Chlorate Ions in a Matrix of Excess Hypochlorite Ions

To adapt the method for determining low concentrations of chlorates in real solutions in the matrix of 0.5-1.2 g/L NaClO, a standard addition of NaClO_3_ was added to the samples of standard solution of NaClO.

It should be noted that chlorites were not found in the solutions of sodium hypochlorite for medicine and veterinary medicine purposes, when using the electrochemical synthesis on the tin dioxide and titanium dioxide anodes doped with platinum group metals. This is probably because of a rapid reaction in the presence of hypochlorite excess [18]:

(6) $$\begin{array}{c} ClO_{2}^{-}+ClO^{-}\to Cl^{-}+ClO_{3}^{-}. \end{array}$$

Therefore, in the procedure under development the determination of chlorites was not considered.

The determination of sodium hypochlorite concentration in the aliquot V_al_ = 2 mL was carried out by indirect titration of formed I_3_^–^ with sodium thiosulfate (0,0025 N) in 10 mL of acetate buffer (рН 3.7-4.2) with a potentiometric fixation of the equivalence point volume (V_1_). A blank titration did not show any improvement of the accuracy of determining hypochlorite.

The determination of NaClO_3_ in another aliquot V_al_ = 2 mL was conducted according to the method described in Section 3.1 after adding 10.0 mL of concentrated hydrochloric acid (10-11 M) and 0.5 g of KBr with a blank titration correction. Actually in this conditions we titrated the sum of ClO^–^ and ClO_3_^–^ and found equivalence point V_2_.

Concentrations (in mg/L) were calculated using equations:

(7) CNaClO=V1·CNa2S2O3·M1/2NaClO·103Val,

(8) CNaClO3=V2−V1·CNa2S2O3−nimp·M1/6NaClO3·103Val,

where V_1_ is the equivalence point volume of sodium thiosulfate for the titration of the sodium hypochlorite in aliquot V_al_; V_2_ is the equivalence point volume of sodium thiosulfate for the titration the sum of sodium hypochlorite and sodium chlorate in the different aliquot of the same volume V_al_; *M*(1/2*NaClO*) = 37.25 g/mol; *M*(1/6*NaClO*_3_) = 17.75 g/mol; n_imp_ is the equivalent amount of impurities, which is determined by blank titration, mmol.

The results of titrations of NaClO solution and NaClO_3_ added to NaClO solution are presented in Table 3. As can be seen from the data, the accuracy of the determination of hypochlorite ions is satisfactory. However, the accuracy of determination of chlorate ions is insufficient. When the concentration of the NaClO_3_ standard samples was 30.0 mg/L, a relative standard deviation S_r_ was found to be 5,8%, and when it was diluted to 15.0 mg/L S_r_ it increased to 10.5%.

When the concentration of sodium hypochlorite in the solution is 1050 mg/L and amount of sodium chlorate is 10 mg/L, then 2.00 mL aliquot requires V_1_ = 22.6 mL and V_2_ = 22.9 mL of 0.0025 N solution of sodium thiosulfate. As follows from the example, the difference in equivalence point volumes (V_2_ - V_1_) is only 0.3 mL. Taking into account the fact that the titration was carried out with the help of a buret (25.0±0.1) mL, a rather large error in determining low concentrations of chlorates occurs. Thus, to determine the concentration of sodium chlorate in the matrix of a large excess of hypochlorite and in order to increase the sensitivity of the method, it is necessary to use a lower-concentrated titrant.

Using a low-concentrated titrant to increase the (V_2_ - V_1_) value becomes possible if 80-95% of hypochlorite ions are removed from the initial solution, depending on the initial concentration. Then burets of less volume with less absolute error can be used increasing the accuracy of the determination of chlorate. The removal of hypochlorite excess from the sample is possible with the help of sodium sulphite:

(9) $$\begin{array}{c} ClO_{}^{-}+SO_{3}^{2-}\to Cl^{-}+SO_{4}^{2-}. \end{array}$$

The authors of [14] showed that at pH 10.5 the reaction proceeds quantitatively, and sodium sulfite does not react with ClO_2_^−^ and ClO_3_^−^ ions, which makes it a selective reagent for ClO^−^. As a result of the reaction, Cl^–^ and SO_4_^2–^ are formed. They are inactive in the iodometric determination of oxygen-containing chlorine derivatives.

We have developed the procedure that realizes reaction (9) to remove excess of hypochlorite ions.

*Procedure*. The concentration of NaClO in the sample solution is determined according to the standard iodometric method in acetate buffer medium at pH 3.5-4.3. Then 10.0 mL aliquot (V_0_) of the sample is transferred into 50 mL flask using pipette (10.0±0.1) mL; 0.4-0.6 mL (V_NaOH_) of 0.05 M NaOH solution is added to a pH of 10.5±0.5 with a pipette (1.00±0.02) mL. The volume of 0.05-0.10 N Na_2_SO_3_ solution needed to reduce the excess sodium hypochlorite is calculated and added to the flask with a pipette (5.00±0.05) mL (*V*_*S*_). The flask is kept closed for 3-5 minutes to complete reaction (9). From the solution, containing 5-10% of the hypochlorite ions from the initial content, two aliquots (V_al_) of 2.00 mL are pipetted (2.00±0.02) mL. The first aliquot is added to 10 mL of acetate buffer (рН 3.7-4.2) and remaining hypochlorite ions are titrated with 0.0010 N sodium thiosulfate to determine the equivalence point volume V_1_. The second aliquot is titrated with 0.0010 N sodium thiosulfate according to the described above procedure for determining the sum of ClO^−^ and ClO_3_^−^ giving the equivalence point volume V_2_. Both titrations are performed using a buret (10.00±0.05) mL. The concentration of sodium chlorate is calculated according to the formula:

(10) CNaClO3=M16NaClO3·V0+VS+VNaOHV0·V2−V1·CNa2S2O3−nimpVal·103.

It is important that in this method there is no need to standardize the sodium sulfite solution. If the content of sodium hypochlorite in the analyzed solution is 500-1200 mg/L, then a solution of Na_2_SO_3_ with a concentration C(Na_2_SO_3_) of 0.05-0.10 N is used. The amount of sodium sulfite should be enough to leave 80-120 mg/L of NaClO after reduction. The volume of this solution (V_S,0_, mL), which is needed for the reduction of ClO^−^ to leave X mg/L of NaClO, is calculated by the formula:

(11) $$\begin{array}{c} V_{S,0}=\frac{\left(C\left(NaClO\right)-X\right)\cdot V_{0}}{M\left(1/2NaClO\right)\cdot C\left(Na_{2}SO_{3}\right)\cdot 10^{3}}, \end{array}$$

At that, any rounded volume V_S_, taking into account absolute error of a pipette, can be added to an aliquot of analyzed solution, but not accurate calculated volume V_S,0_.

For validation of the proposed procedure, a standard solution containing 1025 mg/L of NaClO and 5 mg/L of NaClO_3_ was prepared. It was shown that the maximum accuracy of determination of chlorate in a matrix of 1000 mg/L of NaClO was achieved when more than 75% of hypochlorite ions were reduced (Table 4). As follows from the analysis of the obtained data, even at 77% reduction of hypochlorite ions, the error in determining the content of chlorate ions decreased from 52% to 5%. If the NaClO remaining concentration is 40-60 mg/L and the NaClO_3_ content is less than 20 mg/L, then the titrant should be delivered from the buret (5.00±0.02) mL to increase the accuracy of chlorate determination.

Due to the fact that sodium hypochlorite solutions almost always contain Cl^–^ impurity in various concentrations, the influence of chloride ions on the accuracy of determination of hypochlorite and chlorate was studied. Various amounts of NaCl were introduced into the initial standard solution. It has been shown that the presence of NaCl up to 100 g/L does not affect the results of the determination of hypochlorite and chlorate.

The proposed method was tested on a commercial sodium hypochlorite solution for veterinary purposes VetOx-1000, which is produced by the German-Ukrainian research and production company “BROVAPHARMA” LTD. As mentioned above, for high-purity medical and veterinary preparations based on sodium hypochlorite the absence or minimum content of NaClO_3_ impurity is important. The specified NaClO content in the information about the drug is 1.2±0.1 mg/mL; however, there is no information about the content of chlorate. Five VetOx-1000 solutions from various batches were studied. The results of the analysis are given in Table 5. It shows that the content of NaClO in all samples is within the limits stated by the manufacturer. The concentration of sodium chlorate does not exceed 10 mg/L. For the sample 5 sell-by date had expired by the time of analysis which explains the somewhat higher NaClO_3_ content.

### 3.3. Evaluation of the Instrumental Uncertainty of the Method

Due to the large number of volume measurement operations, it is obvious that the instrumental error contributes a great deal to the total error of the developed procedure. Instrumental errors of different methods of chlorate determination were evaluated as an expanded uncertainty of indirect method of measurement according to [19].

The procedure consists of finding the values of the partial derivatives of concentration *dC*(*NaClO*_3_)/*dx*_*i*_, where *x*_*i*_ are the values that introduce an error in the result. The substitution of the nominal values of the measured quantities into expressions for the partial derivatives gives the sensitivity coefficients *c*_*i*_ of the corresponding input values in the total uncertainty. Finally, the value of the expanded uncertainty (interval of the value distribution) is calculated taking into account the certified tolerance of the glassware and the corresponding distribution law.

We evaluated an uncertainty of the method for the case of analysis of the solution containing 1050 mg/L of sodium hypochlorite solution and 8 mg/L of sodium chlorate.

(1) Procedure without removing excess hypochlorite: here we estimated the uncertainty for the titration of hypochlorite and chlorate with sodium thiosulfate of concentration C(Na_2_S_2_O_3_) = 0.10 N. The volume of the aliquots V_a,1_ = V_a,2_ = 2.00 mL is delivered with a pipette (2.00±0.02) mL; volumes of the equivalence points are to be V_1_ = 0.62 mL and V_2_ = 0.64 mL with use of a buret (1.00±0.02) mL.

The chlorate concentration is calculated by the formula:

(12) CNaClO3=M16NaClO3·CNa2S2O3V2Va,2−V1Va,1,

The expression for the expanded uncertainty is

(13) $$\begin{array}{c} U_{P}=k_{P}\cdot \sqrt{c{\left(V_{1}\right)}^{2}\cdot {\left(\frac{\Delta_{1}}{\sqrt{6}}\right)}^{2}+c{\left(V_{a,1}\right)}^{2}\cdot {\left(\frac{\Delta_{a,1}}{\sqrt{6}}\right)}^{2}+c{\left(V_{2}\right)}^{2}\cdot {\left(\frac{\Delta_{2}}{\sqrt{6}}\right)}^{2}+c{\left(V_{a,2}\right)}^{2}\cdot {\left(\frac{\Delta_{a,2}}{\sqrt{6}}\right)}^{2}}, \end{array}$$

where ∆_1_=∆_2_=0,02 mL; ∆_a,1_=∆_a,2_=0,02 mL; *k*_*P*_ = 1.96.

We assumed that distribution of results of measuring the volume is described by triangular law ($\sigma =\Delta /\sqrt{6}$), whereas the distribution of the resulting value of concentration is normal.

The estimated value of the expanded uncertainty is U_P_ = 20.8 mg/L. Thus, a titration with 0.10 N titrant cannot be used to determine low concentrations of chlorates.

(2) The procedure is similar to the previous one; however, hypochlorite and chlorate are titrated with sodium thiosulfate of lower concentration C (Na_2_S_2_O_3_) = 0.0025 N; 2,00 mL aliquot is delivered with (2.00±0.02) ml pipette. The titrant is delivered with (25.0±0.1) mL buret, and V_1_ = 22.6 mL; V_2_ = 22.9 mL.

The expanded uncertainty is calculated from (13), with ∆_1_ = ∆_2_ = 0.1 mL; ∆_a,1_ = ∆_a,2_ = 0.02 mL. The use of the titrant with lower concentration leads to an almost threefold increase in the accuracy of determination of sodium chlorate U_P_ = 6,4 mg/L. However, the implementation of this procedure also does not provide the necessary accuracy of analysis.

(3) Removal of approximately 90% of hypochlorite enables to use an even less concentrated titrant, titration with the buret of higher measurement accuracy, and to obtain a large difference in the volumes V_2_-V_1_. Concentration of the titrant is C (Na_2_S_2_O_3_) = 0.0010 N, the 2,00 mL aliquot is pipetted with tolerance ±0.02 mL. The equivalence point volumes V_1_ = 3.00 mL and V_2_ = 3.95 mL are delivered with (10.00±0.05) mL buret. The initial solution is sampled with (10.0±0.1) mL pipette (V_0_). The solution of sodium sulfite is added with (5.00±0.05) mL pipette (V_S_). And (10.0±0.1) mL pipette is used for alkaline solution (V_OH_). The chlorate concentration is calculated by the formula:

(14) CNaClO3=M16NaClO3·CNa2S2O3·V0+VS+VOHV0·V2Va,2−V1Va,1

For the expanded uncertainty in this case we use the expression:

(15) UP=kPCV12·Δ162+CVa,12·Δa,162+CV22·Δ262+CVa,22·Δa,262+CV02·Δ062+CVS2·ΔS62+CVOH2·ΔOH62,

where ∆_1_=∆_2_=0,05 mL; ∆_a,1_=∆_a,2_=0,02 mL; ∆_0_=0,1 mL; ∆_S_=0,05 mL; ∆_OH_=0,01 mL; *k*_*P*_ = 1.96.

The performed calculation shows that the main contribution to the total uncertainty of the chlorate concentration is caused by the uncertainty of sampling aliquots and measuring the equivalence point volumes. The uncertainties of the addition of alkaline solutions and sodium sulfite to remove excess sodium hypochlorite are negligibly small. The contribution of instrumental uncertainty arising during the preparation of 0.0010 N titrant from 0.10 N solution was evaluated separately. It is also insignificant.

The procedure with removal of excess hypochlorite gives the expanded uncertainty of chlorate determination U_P_ = 0.8 mg/L.

If titration is carried out with the help of a buret (5.00±0.02) mL (∆_1_ = ∆_2_ = 0.02 mL), then we obtain even more accurate determination of NaClO_3_ with U_P_ = 0.6 mg/L.

## 4. Conclusions

It is shown that in order to increase the accuracy of the determination of microquantities of chlorates in a matrix of a large excess of hypochlorite it is advisable to use blank titration, since in this case possible systematic errors due to the presence of impurities of oxidants or reducing agents in the reagents are corrected. For the quantitative determination of low concentrations of chlorates, we proposed to remove 85-95% of hypochlorite ions by reducing their excess with sodium sulfite at pH 10.5. The solution of sodium sulfite does not require standardization in the proposed procedure. The possibility of quantitative determination of chlorate ions in amounts of 2-50 mg/L in the presence of 50-500-fold excess of sodium hypochlorite with an error of 5% was experimentally confirmed.

## Figures and Tables

**Table 1:** Determination of sodium chlorate in the presence of potassium bromide (N = 5 is the number of titrations of the same sample and P = 0.95 is the confidence interval).

*C*(NaClO_3_), mg/L		*S*_r_, %
*Standard solution*	*Results of titration*	
30,0	29,0±3,1	12,2
60,0	59,2±3,8	7,3
120,0	118,0±5,5	5,3

**Table 2:** Determination of sodium chlorate content without and with reagent blank correction (N = 5, P = 0.95).

*C*(NaClO_3_), mg/L			*S*_r_, %
*Standard solution*	*Results of titration without blank correction*	*Results of titration with blank correction*	
30,0	34,2±3,0	30,1±1,2	4.6
60,0	65,2±3,6	61,5±2,2	4.1
120,0	126,0±4,1	120,8±3,1	2.9

**Table 3:** The results of the determination of sodium hypochlorite and sodium chlorate (N = 5, P = 0.95).

NaClO solution	*C*(NaClO),	*S*_r_, %	*C*(NaClO_3_), mg/L		*S*_r_, %
	mg/L		*Standard addition*	*Result of analysis*	
Initial solution	1025.0±4.6	0.51	30.0	27.7±1.4	5.8
Initial solution 2-fold diluted with distilled water	508.9±4.3	0.96	15.0	14.1±1.3	10.5

**Table 4:** The results of the determination of NaClO_3_ in the standard solution of 1025 mg/L NaClO with 5 mg/L of NaClO_3_ according to the procedure with the reduction of ClO^−^ excess with 0.085 N Na_2_SO_3_; V_0_ = 10 mL; V_NaOH_ = 0.5 mL; (N = 5, P = 0.95).

No.	Added Na_2_SO_3_(*V*_*S*_), mL	Calculated according to (7) NaClO, mg/L	Reduced NaClO, %	Calculated according to (10) NaClO_3_, mg/L	*S*_r_, %
1	0	1024±4,7	0	3,7±1,7	52,4
2	2,0	391±4,1	61,8	4,1±1,0	27,8
3	2,5	233±3,6	77,3	4,8±0,3	7,1
4	2,8	138±3,5	86,5	4,9±0,2	4,7
5	3,0	75±3,3	92,7	4,9±0,2	4,7

**Table 5:** The results of the determination of sodium hypochlorite and sodium chlorate in VetOx-1000 (N = 5, P = 0.95).

VetOx-1000	*C*(NaClO), mg/L	*S*_r_, %	С(NaClO_3_), mg/L	*S*_r_, %
1	1267±4,7	0,42	4,3±0.2	5,3
2	1218±6,5	0,61	5,5±0.3	6,2
3	1231±5,5	0,51	3,5±0.2	6,5
4	1205±4,8	0,45	9.2±0.4	4,9
5	1105±3,5	0,36	22.1±0.5	2,6

## Data Availability

The data used to support the findings of this study are included within the article.

## References

[1] Wang L., Bassiri M., Najafi R. (2007). Hypochlorous acid as a potential wound care agent. Part I. Stabilized hypochlorous acid: a component of the inorganic armamentarium of innate immunity. *Journal of Burns and Wounds*.

[2] Galenko-Yaroshevskii P. A., Gumenyuk S. E., Pavlenko S. G. (1999). Prolonged bactericidal effect of sodium hypochlorite. *Bulletin of Experimental Biology and Medicine*.

[3] Kim H. J., Lee J.-G., Kang J. W. (2008). Effects of a low concentration hypochlorous acid nasal irrigation solution on bacteria, fungi, and virus. *The Laryngoscope*.

[4] Neodo S., Rosestolato D., Ferro S., De Battisti A. (2012). On the electrolysis of dilute chloride solutions: Influence of the electrode material on Faradaic efficiency for active chlorine, chlorate and perchlorate. *Electrochimica Acta*.

[5] Sizeneva I. P., Kondrashova N. B., Val'tsifer V. A. (2005). Spontaneous decomposition of industrially manufactured sodium hypochlorite solutions. *Russian Journal of Applied Chemistry*.

[6] Feretti D., Zerbini I., Ceretti E. (2008). Evaluation of chlorite and chlorate genotoxicity using plant bioassays and in vitro DNA damage tests. *Water Research*.

[7] Steffen C., Wetzel E. (1993). Chlorate poisoning: mechanism of toxicity. *Toxicology*.

[8] Mccauley P. T., Robinson M., Daniel F. B., Olson G. R. (1995). The effects of subchronic chlorate exposure in Sprague-dawley rats. *Drug and Chemical Toxicology*.

[9] Vogel A. I., Mendham J., Denney R. C., Barnes J. D. (2000). *Vogel's Textbook of Quantitative Chemical Analysis*.

[10] Garcia-Villanova R. J., Oliveira Dantas Leite M. V., Hernández Hierro J. M., de Castro Alfageme S., García Hernández C. (2010). Occurrence of bromate, chlorite and chlorate in drinking waters disinfected with hypochlorite reagents. Tracing their origins. *Science of the Total Environment*.

[11] Hosseini S. G., Pourmortazavi S. M., Gholivand K. (2009). Spectrophotometric determination of chlorate ions in drinking water. *Desalination*.

[12] Thiagarajan S., Wu Z.-Y., Chen S.-M. (2011). Amperometric determination of sodium hypochlorite at poly MnTAPP-nano Au film modified electrode. *Journal of Electroanalytical Chemistry*.

[13] Tang T.-F., Gordon G. (1980). Quantitative determination of chloride, chlorite, and chlorate ions in a mixture by successive potentiometric titrations. *Analytical Chemistry*.

[14] Adam L. C., Gordon G. (1995). Direct and sequential potentiometric determination of hypochlorite, chlorite, and chlorate ions when hypochlorite ion is present in large excess. *Analytical Chemistry*.

[15] Ikeda Y., Tang T.-F., Gordon G. (1984). Iodometric method for determination of trace chlorate ion. *Analytical Chemistry*.

[16] Brauer G. (1963). *Handbook of Preparative Inorganic Chemistry*.

[17] Rice E. W., Baird R. B., Eaton A. D. (2012). *Standard Methods for the Examination of Water and Wastewater*.

[18] Siddiqui M. S. (1996). Chlorine-ozone interactions: Formation of chlorate. *Water Research*.

[19] *Evaluation of measurement data – Guide to the expression of uncertainty in measurement JCGM 100:2008 (GUM 1995 with minor corrections), Paris: BIPM Joint Committee for Guides in Metrology, 2008*.

